# Maternal dietary consumption of legumes, vegetables and fruit during pregnancy, does it protect against small for gestational age?

**DOI:** 10.1186/s12884-018-2123-4

**Published:** 2018-12-11

**Authors:** Juan Miguel Martínez-Galiano, Carmen Amezcua-Prieto, Inmaculada Salcedo-Bellido, Guadalupe González-Mata, Aurora Bueno-Cavanillas, Miguel Delgado-Rodríguez

**Affiliations:** 1 Department of Health Sciences, University of Jaen, Campus de Las Lagunillas s n Edificio B3, 23071 Jaén, Spain; 2 CIBER de Epidemiología y Salud Pública (CIBERESP), Jaén, Spain; 3 Department of Preventive Medicine and Public Health, University of Granada, Granada, Spain; 4 Biosanitary Research Institute Granada (IBS-Granada), Granada, Spain

**Keywords:** Small for gestational age, Maternal diet, Fruits, Legumes, Vegetables, Selenium

## Abstract

**Background:**

Different diets during pregnancy might have an impact on the health, reflected in the birthweight of newborns. The consumption of fruits and vegetables during pregnancy and the relationship with newborn health status have been studied by several authors. However, these studies have shown inconsistent results. Purpose: We assessed whether certain foods were related to the risk of small for gestational age (SGA).

**Methods:**

A matched by age (± 2 years) and hospital 1:1 case-control study of 518 pairs of pregnant Spanish women in five hospitals was conducted. The cases were women with an SGA newborn at delivery (neonates weighting less than the 10th percentile, adjusted for gestational age at delivery and sex, were diagnosed as SGA). The control group comprised women giving birth to babies adequate for gestational age (AGA). Mothers who gave birth to babies large for gestational age (LGA) were excluded. Data were gathered concerning demographic characteristics, socioeconomic status, toxic habits and diet. A food frequency questionnaire (FFQ) comprising 137 items was completed by all participants. The intake of vegetables, legumes and fruits was categorized in quintiles (Q1–Q5). Crude values and and adjusted odds ratios (AORs) and 95% confidence intervals (CIs) were estimated using conditional logistic regression. The variables for adjustment were as follows: preeclampsia, education, smoking, weight gain per week during pregnancy, fish intake and previous preterm/low birthweight newborns.

**Results:**

Total pulse intake showed an inverse association with the risk of SGA (trend *p* = 0.02). Women with an intake of fruits above 420 g/day (Q5), compared with women in Q1 (≤ 121 g/day) showed a decreased risk of SGA (AOR = 0.63, 95% CI = 0.40–0.98). The total consumption of vegetables was not associated with the risk of SGA. The intake of selenium was assessed: a protective association was observed for Q3–5; a daily intake above 60 μg was associated with a lower risk of SGA (AOR = 0.39, 95% CI: 0.22–0.69).

**Conclusions:**

Fruits, pulses and selenium reduce the risk of SGA in Spanish women.

**Electronic supplementary material:**

The online version of this article (10.1186/s12884-018-2123-4) contains supplementary material, which is available to authorized users.

## Background

Small for gestational age (SGA) newborns are defined as those with a birthweight in the <10th percentile according to gestational age [1]. There are important adverse consequences for SGA newborns, including higher mortality and psychic, physical and social health problems in the short, medium and long term [2–8]. Between 5.3% and 23.7% of live newborns are SGA [2, 8–10].

Several risk factors for SGA have been identified. Among the non-dietary variables, the main factors are maternal education, income, smoking, body mass index (BMI), low weight gain during pregnancy, diseases during pregnancy (anaemia, preeclampsia, hypertension, infections, etc.), previous low birthweight and factors related to preterm delivery, inter alia [9–15]. In this regard, maternal diet may play an important role. Among others, it may have an “additional” role in various risks during pregnancy, such as gestational diabetes, maternal hypertension/preeclampsia, preterm birth and foetal growth restriction [16, 17].

The consumption of fruits and vegetables during pregnancy and the relationship with newborn health status has been studied by several authors [18–20]. Previous studies show inconsistent results. In the *INfancia and Medio Ambiente* (INfancy and Environment [INMA]) cohort study undertaken in Spain, incorporating 787 pregnant women with 11.5% SGA, a high intake of vegetables during pregnancy showed a lower incidence of SGA; no association was observed for fruit consumption [18]. In a cohort study in Denmark with 43,585 women) [19], the results contradicted those of the INMA cohort; that is, maternal consumption of fruit, but not vegetables, during pregnancy was associated with increased birthweight. A systematic review that did not include meta-analysis found no evidence that the intake of fruits and vegetables during pregnancy reduced the risk of having a SGA newborn [20].

The negative association of the consumption of fruits and vegetables with the risk of SGA can be explained by the high content of vitamins and micronutrients. Some studies have reported an inverse association of different vitamins (D, folic acid, etc.) and the risk of SGA [21, 22]. Several studies have analysed the relationship between selenium and newborn size, also with conflicting results [23–27]. Selenium has been implied to influence inflammation, oxidation and the proper functioning of the immunological system [28].

The intake of legumes is very common in Spain and other Mediterranean countries, as these are key elements of the Mediterranean diet. However, there are no published studies relating legume consumption to the risk of SGA. Given that previous studies correlating vegetable and fruit consumption with the risk of SGA present conflicting results, the aim of this study is to provide a fresh assessment of the effect of the intake of vegetables, legumes and fruits on the risk of SGA in a Southern European population.

## Methods

The study population included women attending five hospitals in Eastern Andalusia (Spain): the University of Jaen Hospital (UJH), Ubeda Hospital (UB), the University of Granada Hospitals ([UGH] two centres) and Poniente Hospital (PH), serving 1.8 million people. Case and control groups were collected from 15 May 2012 to 15 July 2015.

Ethical approval was obtained from the Ethics Committees of the hospitals participating in the study: Comité de Ética de la Investigación del Complejo Hospitalario Universitario de Granada (Committee of Ethics of Investigation of the UGH), Comité de Ética de la Investigación del Hospital de Poniente (the Committee of Ethics of Investigation of the PH), Comité de Ética de la Investigación del Complejo Hospitalario Universitario de Jaén; (the Committee of Ethics of Investigation of the UJH) and Comité de Ética de la Investigación del Hospita de Úbeda (the Committee of Ethics of Investigation of the UB). Informed consent was obtained from the women participating in the study and we followed the protocols established by the respective health centres for accessing data from medical records to carry out this type of research with the purpose of publication/disclosure to the scientific community.

We estimated the appropriate sample size based on the results of a similar study [29]. To detect a significant (*p* <  0.05, odds ratio [OR] = 0.6) difference between extreme quintiles with a statistical power of 80%, we estimated that 447 pairs of cases and controls were required.

### Cases

The eligibility criteria for cases were the delivery of a single live newborn diagnosed as small for gestational age (SGA) according to the tables developed for the Spanish population [30], without congenital malformations, during the study period and resident in the referral area of the hospital. Nineteen women declined participation. A total of 533 cases were selected: 79 (UJH), 369 (UGH), 46 (UB) and 39 (PH).

### Controls

A pair of newborns matched by age at delivery (± 2 years) was selected within a week of inclusion of a case at the same hospital. Eligible women were those with a newborn of the appropriate gestational age meeting the same inclusion criteria for cases (residence in the referral area of the hospital and no malformations); women with large for gestational age (LGA) newborns were excluded. Sixty-five women declined participation.

### Data collection

Data were gathered through personal interviews (conducted in the hospital by a specifically trained midwife within two days of delivery), clinical charts and prenatal care records. Information was obtained for the following variables: mother’s vital data (age at pregnancy, race, pre-pregnancy body mass index [BMI], educational level, marital status, income level and occupation), obstetric history (parity and abortions), previous adverse perinatal outcomes, conditions during pregnancy (infections, preeclampsia, diabetes and other obstetric conditions), birth weight (weight in grams in the delivery room), prescribed and over-the-counter drugs, smoking during pregnancy and prenatal care (number of visits and date of first visit, weight gain during pregnancy). Social class was coded according to five main levels (ranging from I [the highest] to V [the lowest]) based on the classification of the Spanish Society of Epidemiology [31], which is close to that of the Black Report [32]. Utilization of prenatal care was measured using the Kessner Index [33].

Alcohol consumption during and before pregnancy was assessed using a structured questionnaire in which the number and types of drinks on weekdays, weekends (including Friday evenings), and holidays (including the eve) were recorded.

### Dietary assessment

The baseline questionnaire (Additional file 1) included a semiquantitative food frequency questionnaire, previously validated in Spain, comprising 137 items and open-ended questions to obtain information on use of dietary supplements [34]. The questionnaire was based on typical portion sizes and provided 9 options for the frequency of intake in the previous year for each food item (ranging from “never” or “almost never” to “≥ 6 times/day”). A dietician updated the nutrient data bank using the latest available information included in the food composition tables for Spain [35, 36]. After computing total energy intake, a total of 15 matched pairs were excluded because of unreliable dietary assessment (total energy intake > 4000 Kcal/day), leaving 518 pairs for analysis.

### Statistical analysis

Food and nutrient intake were adjusted for total energy intake using the residuals method and separate regression models were performed to obtain the residuals [37]. Energy-adjusted food and nutrient intake were categorized in quintiles. It is recommended that selenium intake in pregnancy be at least 60 μg/day [38] and this cut-off level was also applied in the analysis of this micronutrient. Odds ratios (ORs) and 95% confidence intervals (CIs) were estimated with conditional regression logistic models, with adjustments for potential confounders (adjusted ORs [aORs]): preeclampsia, education, smoking, weight gain per week during pregnancy, fish intake and previous preterm/low birthweight newborns. The addition of other variables (such as BMI) did not change the coefficients of the models appreciably (< 10% change). All *p-*values are 2-tailed. Statistical significance was set at *p* <  0.05. Analyses were performed in the Stata 14 program (College Station, TX).

## Results

In all, 1036 women – 518 cases and 518 controls – participated in the study. The description of the study population is shown in Table 1. Maternal marital status influences the risk of SGA (*p* = 0.036). Well-known risk factors for SGA showed significant relationships in our study population, such as maternal smoking, previous low birth weight and/or preterm delivery, maternal BMI, low weight gain during pregnancy, preeclampsia and fish intake. The duration of gestation was shorter in the cases than in the controls and the frequency of Caesarean section was similar in both groups.

**Table 1:** Description of the study population

Variable	Cases	Controls	*P*-value
Marital status, n (%)			0.036
Single	37 (7.1)	42 (8.1)	
Stable couple	161 (31.1)	124 (23.9)	
Married	320 (61.8)	352 (68.0)	
Education level, n (%)			0.084
Primary	112 (21.6)	93 (17.9)	
High school, not ended	42 (8.1)	28 (5.4)	
High school	185 (35.7)	190 (36.7)	
University	179 (34.6)	207 (40.0)	
Previous preterm/low birthweight newborn (yes), n (%)	64 (12.4)	26 (5.0)	< 0.001
Kessner index (prenatal care), n (%)			0.737
Adequate	259 (50.0)	253 (48.8)	
Intermediate	185 (35.7)	182 (35.2)	
Inadequate	74 (14.3)	83 (16.0)	
Smoking during pregnancy, n (%)	149 (28.8)	80 (15.4)	< 0.001
Preeclampsia, n (%)	46 (8.9)	11 (2.1)	< 0.001
Weight gain throughout pregnancy (g/week), mean (SD)	278 (121)	310 (114)	< 0.001
Body mass index in the first trimester of pregnancy (Kg/m^2^), mean (SD)	23.1 (4.5)	23.9 (4.1)	< 0.001
Alcohol intake (g/week), mean (SD)	4.2 (18.5)	3.1 (15.2)	0.312
Fish intake (g/day), mean (SD)	83.4 (48.4)	92.0 (57.0)	0.009
Gestational age (days), mean (SD)	271 (11)	276 (9)	< 0.001
Delivery by caesarean, n (%)	127 (24.2)	120 (23.2)	0.854

The relationship between legume intake and risk of SGA is shown in Table 2. In general, the highest levels of consumption are associated with a lower risk of SGA, although statistical significance is only achieved for kidney beans (aOR = 0.67, 95% CI: 0.48–0.94). The total amount of legumes was also analysed in quintiles. Significant *p*-values for the trend can be observed in both the crude and adjusted analyses: the higher the intake, the lower the risk.

**Table 2:** Frequency of intake of different legumes and risk of SGA

Frequency of intake	Casesn (%)	Controlsn (%)	OR (95% CI)	aOR^a^ (95% CI)
Lentils				
Never	21 (4.1)	19 (3.7)	1 (ref.)	1 (ref.)
1–3 times per month	81 (15.6)	85 (16.4)	0.85 (0.43–1.68)	0.81 (0.38–1.71)
Once per week	406 (78.4)	396 (76.5)	0.91 (0.49–1.70)	0.81 (0.41–1.60)
> Once per week	10 (1.9)	18 (3.5)	0.50 (0.19–1.35)	0.41 (0.14–1.22)
Kidney beans				
Never	134 (25.9)	106 (20.5)	1 (ref.)	1 (ref.)
1–3 times per month	145 (28.0)	141 (27.2)	0.80 (0.57–1.14)	0.84 (0.57–1.23)
≥ Once+ per week	239 (46.1)	271 (52.3)	0.70 (0.51–0.95)	0.67 (0.48–0.94)
Chickpeas				
Never	52 (10.0)	46 (8.9)	1 (ref.)	1 (ref.)
1–3 times per month	129 (24.9)	140 (24.9)	0.81 (0.51–1.30)	0.79 (0.47–1.31)
≥ Once per week	337 (65.1)	332 (64.1)	0.89 (0.58–1.39)	0.83 (0.51–1.33)
Peas				
Never	170 (32.8)	152 (29.3)	1 (ref.)	1 (ref.)
1–3 times per month	184 (35.5)	188 (36.3)	0.86 (0.63–1.16)	0.84 (0.61–1.18)
Once per week	148 (28.6)	158 (30.5)	0.83 (0.60–1.14)	0.78 (0.55–1.10)
> Once per week	16 (3.1)	20 (3.9)	0.69 (0.34–1.41)	0.59 (0.27–1.28)
Legumes, g/day in quintiles				
Q1 (≤ 16.4 g/d)	112 (21.6)	104 (20.1)	1 (ref.)	1 (ref.)
Q2 (16.5–23.6)	115 (22.2)	104 (20.1)	1.04 (0.72–1.54)	1.07 (0.70–1.64)
Q3 (23.7–28.3)	115 (22.2)	103 (19.9)	1.05 (0.71–1.56)	1.16 (0.76–1.77)
Q4 (28.4–33.0)	97 (18.7)	104 (20.1)	0.89 (0.60–1.32)	0.88 (0.58–1.36)
Q5 (> 33.0)	79 (15.3)	103 (19.9)	0.72 (0.49–1.07)	0.68 (0.44–1.04)
P for trend			0.050	0.020

^a^ Adjusted for preeclampsia, education, smoking, gain weight per week during pregnancy, fish intake and previous preterm/low birthweight newborn

Women with an intake of fruits > 420 g/day (Q5) compared with those with an intake ≤121 g/day (Q1) showed a decreased risk of SGA (aOR = 0.63, 95% CI = 0.40–0.98), although the trend was not significant (*p* for the trend = 0.136) (Table 3). Regarding the consumption of specific fruits, only melon (an intake of two or more times a week vs no consumption) yielded a significant relationship (aOR = 0.48, 95% CI = 0.29–0.78).

**Table 3:** Frequency of intake of different fruits and risk of SGA

Frequency of intake	Casesn (%)	Controls n (%)	OR (95% CI)	aOR^a^ (95% CI)
Orange				
Never	50 (9.7)	36 (7.0)	1 (ref.)	1 (ref.)
vs. ≥ Once per day	85 (16.4)	91 (17.6)	0.67 (0.40–1.13)	0.68 (0.38–1.21)
Banana				
Never	78 (15.1)	70 (13.5)	1 (ref.)	1 (ref.)
vs. ≥ 5 times per week	33 (1.4)	47 (9.1)	0.64 (0.37–1.11)	0.60 (0.32–1.11)
Apple				
Never	60 (11.6)	51 (9.9)	1 (ref.)	1 (ref.)
vs. ≥ 5 times per week	46 (7.7)	46 (8.9)	0.74 (0.42–1.30)	0.75 (0.41–1.39)
Strawberry				
Never	108 (20.9)	100 (19.3)	1 (ref.)	1 (ref.)
vs. ≥ 2 times per week	56 (10.8)	53 (10.2)	0.97 (0.61–1.53)	0.88 (0.54–1.45)
Cherry				
Never	140 (27.0)	123 (23.8)	1 (ref.)	1 (ref.)
vs. ≥ 2 times per week	39 (7.5)	49 (9.5)	0.69 (0.42–1.12)	0.63 (0.37–1.08)
Peach				
Never	111 (21.4)	94 (18.2)	1 (ref.)	1 (ref.)
vs. ≥ 2 times per week	66 (12.7)	78 (15.1)	0.72 (0.46–1.13)	0.79 (0.49–1.29)
Watermelon				
Never	82 (15.8)	69 (13.3)	1 (ref.)	1 (ref.)
vs. ≥ 5 times per week	31 (6.0)	34 (6.6)	0.74 (0.41–1.32)	0.64 (0.33–1.22)
Melon				
Never	108 (20.9)	80 (15.4)	1 (ref.)	1 (ref.)
vs. ≥ 2 times per week	59 (11.4)	82 (15.8)	0.53 (0.34–0.84)	0.48 (0.29–0.78)
Kiwi fruit				
Never	190 (36.7)	186 (35.9)	1 (ref.)	1 (ref.)
vs. ≥ 2 times per week	80 (15.4)	88 (17.0)	0.90 (0.62–1.29)	1.02 (0.68–1.52)
Grapes				
Never	190 (36.7	189 (36.5)	1 (ref.)	1 (ref.)
vs. ≥ 2 times per week	34 (6.6)	34 (6.6)	0.99 (0.60–1.66)	0.87 (0.50–1.50)
Fruit, g/day, quintiles				
Q1 (≤ 121 g/d)	129 (24.9)	104 (20.1)	1 (ref.)	1 (ref.)
Q2 (122–170)	109 (21.0)	104 (20.1)	0.84 (0.58–1.24)	0.87 (0.57–1.35)
Q3 (171–272)	100 (19.3)	103 (19.9)	0.76 (0.52–1.11)	0.73 (0.48–1.12)
Q4 (273–420)	98 (18.9)	104 (20.1)	0.74 (0.51–1.09)	0.73 (0.47–1.13)
Q5 (> 420)	82 (15.8)	103 (19.9)	0.63 (0.42–0.93)	0.63 (0.40–0.98)
P for trend			0.089	0.136

^a^ Adjusted for preeclampsia, education, smoking, body mass index, gain weight per week during pregnancy, fish intake and previous preterm/low birthweight newborn

The consumption of dried fruits and nuts is displayed in Table 4. Total dried fruits showed a lower risk in Q5 (> 3.8 g/day) vs Q1 (< 0.1 g/day) in the crude analysis, although in multivariate analysis the association was borderline (aOR = 0.67, 95% CI: 0.42–1.05. Walnuts and other nuts did not exhibit any association with SGA.

**Table 4:** Frequency of intake of dried fruit and nuts and risk of SGA

Frequency of intake (quintiles, Q)	Casesn (%)	Controls n (%)	OR (95% CI)	aOR^a^ (95% CI)
Dried fruit (g/day)				
Q1 (< 0.1)	126 (24.3)	104 (20.1)	1 (ref.)	1 (ref.)
Q2 (0.1–0.3)	113 (21.8)	104 (20.1)	0.89 (0.61–1.29)	0.88 (0.58–1.34)
Q3 (0.31–1.1)	105 (20.7)	103 (19.9)	0.83 (0.57–1.22)	0.89 (0.58–1.38)
Q4 (1.11–3.8)	91 (17.6)	104 (20.1)	0.70 (0.47–1.03)	0.70 (0.45–1.10)
Q5 (> 3.8)	83 (16.0)	103 (19.9)	0.63 (0.42–0.95)	0.67 (0.42–1.05)
P for trend			0.099	0.193
Walnuts (g/day)				
Q1 (< 0.3)	114 (22.0)	104 (20.1)	1 (ref.)	1 (ref.)
Q2 (0.3–1.4)	99 (19.1)	104 (20.1)	0.86 (0.58–1.27)	0.75 (0.49–1.14)
Q3 (1.41–2.6)	90 (17.4)	103 (19.9)	0.79 (0.54–1.17)	0.78 (0.51–1.21)
Q4 (2.61–5.0)	114 (22.0)	104 (20.1)	1.01 (0.69–1.46)	1.02 (0.68–1.53)
Q5 (> 5.0)	101 (19.5)	103 (19.9)	0.89 (0.60–1.31)	0.83 (0.55–1.27)
P for trend			0.873	0.517
Other nuts^b^ (g/day)				
Q1 (< 0.8)	104 (20.8)	104 (20.1)	1 (ref.)	1 (ref.)
Q2 (0.8–2.0)	83 (16.0)	104 (20.1)	0.79 (0.52–1.17)	0.80 (0.51–1.24)
Q3 (2.01–3.4)	114 (22.0)	103 (19.9)	1.09 (0.74–1.60)	1.07 (0.70–1.64)
Q4 (3.41–5.5)	96 (18.5)	104 (20.1)	0.91 (0.62–1.35)	0.91 (0.59–1.40)
Q5 (> 5.5)	121 (23.4)	103 (19.9)	1.16 (0.80–1.66)	1.21 (0.81–1.81)
P for trend			0.188	0.234

^a^Adjusted for preeclampsia, education, smoking, body mass index, gain weight per week during pregnancy, fish intake and previous preterm/low birthweight newborn

^b^Almond, peanut, pistachio, etc.

No association was found between the total consumption of vegetables and the risk of SGA (Table 5). Regarding specific vegetables, significant associations were observed for asparagus, garlic and green beans, with borderline associations for eggplants, but not for the remaining vegetables. Finally, the consumption of selenium was assessed (Table 6): a protective association was observed for Q3–5. Given that the recommended daily intake is 60 μg, we found that an intake above this level was associated with a lower risk of SGA (aOR = 0.39, 95% CI: 0.22–0.69).

**Table 5:** Frequency of intake of different vegetables and risk of SGA

Frequency of intake	Cases n (%)	Controls n (%)	OR (95% CI)	aOR^a^ (95% CI)
Swiss chard				
Never	128 (24.7)	110 (21.2)	1 (ref.)	1 (ref.)
vs. ≥ 2 times per week	79 (15.3)	72 (13.9)	0.91 (0.60–1.39)	0.88 (0.55–1.39)
Cabbage				
Never	234 (45.2)	220 (42.5)	1 (ref.)	1 (ref.)
vs. ≥ 2 times per week	44 (8.5)	41 (7.9)	1.01 (0.63–1.58)	0.94 (0.57–1.55)
Lettuce				
Never	40 (7.7)	33 (6.4)	1 (ref.)	1 (ref.)
vs. ≥ 5 times per week	141 (27.2)	122 (23.6)	0.94 (0.56–1.60)	0.83 (0.46–1.51)
Tomato				
Never	50 (9.7)	40 (7.7)	1 (ref.)	1 (ref.)
vs. ≥ 5 times per week ek	193 (37.3)	177 (34.2)	0.86 (0.53–1.40)	1.02 (0.60–1.75)
Carrot				
Never	97 (18.7)	68 (13.1)	1 (ref.)	1 (ref.)
vs. ≥ 5 times per week	74 (14.3)	60 (11.6)	0.88 (0.56–1.39)	0.90 (0.55–1.48)
Green bean				
Never	124 (23.9)	90 (17.4)	1 (ref.)	1 (ref.)
vs. ever	398 (76.1)	428 (82.6)	0.66 (0.49–0.90)	0.55 (0.38–0.90)
Eggplants				
Never	71 (13.7)	57 (11.0)	1 (ref.)	1 (ref.)
vs. ≥ 2 times per week	70 (13.5)	87 (16.8)	0.63 (0.38–1.02)	0.59 (0.34–1.01)
Pepper				
Never	102 (19.7)	93 (18.0)	1 (ref.)	1 (ref.)
vs. ≥ 2 times per week	96 (18.5)	105 (20.3)	0.83 (0.56–1.24)	0.77 (0.50–1.20)
Asparagus				
Never	191 (36.9)	163 (31.5)	1 (ref.)	1 (ref.)
vs. ≥ 2 times per week	36 (7.0)	48 (9.3)	0.64 (0.39–1.03)	0.53 (0.31–0.89)
Cold vegetable soup (Spanish ‘gazpacho’)				
Never	196 (37.8)	169 (32.6)	1 (ref.)	1 (ref.)
vs. ≥ 2 times per week	44 (8.5)	41 (7.9)	0.91 (0.58–1.46)	0.75 (0.45–1.24)
Onion				
Never	78 (15.1)	73 (14.1)	1 (ref.)	1 (ref.)
vs. ≥ 5 times per week	159 (30.7)	117 (22.6)	1.32 (0.89–1.96)	1.26 (0.81–1.97)
Garlic				
Never	104 (20.1)	75 (14.5)	1 (ref.)	1 (ref.)
vs. ever	414 (79.9)	443 (75.5)	0.68 (0.49–0.94)	0.67 (0.46–0.97)
Mushrooms				
Never	139 (26.8)	126 (24.3)	1 (ref.)	1 (ref.)
vs. ≥ 2 times per week	36 (7.0)	32 (6.2)	1.01 (0.59–1.74)	0.90 (0.48–1.67)
Total amount of vegetables (g/day), quintiles				
Q1 (≤ 228.9)	122 (23.6)	104 (20.1)	1 (ref.)	1 (ref.)
Q2 (229.0–329.1)	89 (17.2)	104 (20.1)	0.72 (0.49–1.07)	0.75 (0.48–1.15)
Q3 (329.2–432.5)	90 (17.4)	103 (19.9)	0.72 (0.49–1.08)	0.75 (0.48–1.15)
Q4 (432.6–573.0)	96 (18.5)	104 (20.1)	0.77 (0.53–1.13)	0.80 (0.53–1.22)
Q5 (> 573.0)	121 (23.4)	103 (19.9)	0.99 (0.69–1.44)	0.91 (0.60–1.39)
P for trend			0.177	0.401

^a^ Adjusted for preeclampsia, education, smoking, gain weight per week during pregnancy, fish intake and previous preterm/low birthweight newborn

**Table 6:** Association between selenium (μg/day) and risk of SGA

Quintiles	Casesn (%)	Controls n (%)	OR (95% CI)	aOR^a^ (95% CI)
Selenium (μg/day)				
Q1 (≤ 72.25)	132 (25.5)	104 (20.1)	1 (ref.)	1 (ref.)
Q2 (72.26–84.49)	110 (21.4)	104 (20.1)	0.83 (0.57–1.20)	0.73 (0.44–1.22)
Q3 (84.50–96.20)	86 (16.6)	103 (19.9)	0.63 (0.42–0.95)	0.46 (0.27–0.80)
Q4 (96.21–110.63)	94 (18.2)	104 (20.1)	0.71 (0.49–1.03)	0.58 (0.36–0.94)
Q5 (> 110.63)	96 (18.5)	103 (19.9)	0.72 (0.49–1.07)	0.53 (0.32–0.87)
P for trend			0.581	0.594
< 60	65 (12.6)	32 (6.2)	1 (ref.)	1 (ref.)
≥ 60	453 (87.4)	486 (93.8)	0.47 (0.30–0.73)	0.39 (0.22–0.69)

^a^ Adjusted for preeclampsia, education, smoking, body mass index, gain weight per week during pregnancy and previous preterm/low birthweight newborn

## Discussion

The aim of this report was to assess the effect of the intake of vegetables, legumes and fruits on the risk of SGA in a Southern European population. According to our results, the overall consumption of legumes shows an inverse association with the frequency of SGA. The same holds for fruit consumption > 420 g/day. No relationship between the general consumption of vegetables and fruits and SGA was observed, nor for consumption of nuts. A protective association was observed with selenium intake above the recommended levels.

The consumption of fruits in the diet of pregnant women was studied in a case-control study in New Zealand comprising 844 cases (SGA) and 870 controls; it was reported that the insufficient consumption of fruit (intake < 0.75 servings of fruits a day) during pregnancy increased the risk of SGA [39]. Similar conclusions were obtained from a Danish cohort study [19] and an Indian prospective descriptive study carried out over two years [40]. In contrast, in the Spanish INMA cohort study [18], no such relationship was observed. We found an association for Q5 (> 420 g/day), although no significant trend was apparent. This association may be due to the high content of vitamins in fruits [41].

We did not find any significant relationship with dried fruits and nuts. These foods have interesting components in terms of reducing the risk of cardiovascular diseases [42] and they also have a high content of selenium. However, the amount ingested by women in our study was rather small: for Q5 consumption of dried fruits was > 3.8 g/day and of walnuts was > 5 g/day; this latter case implies a single unit per day.

The lack of a relationship between total vegetable intake and SGA in our study agrees with the conclusions of a systematic review [20]. In contrast, several studies have found that vegetable intake increases foetal size. The first of these is the INMA study [18], which reported a beneficial effect on foetal growth attributed by the authors to the high content of antioxidants and folic acid in vegetables. Two other reports, from Denmark [19] and India [40], noted the same results. These studies did not analyse specific types of vegetables and therefore it is not possible to compare our results for green beans, garlic and asparagus (in which protective associations with SGA risk were observed).

Insufficient consumption of selenium increases the frequency of pre-eclampsia, a clear risk factor for SGA [23]. An analysis on a subset (126 pregnant adolescent women) from a prospective observational study in London found low levels of selenium in women delivering an SGA newborn [24]. Sun et al., in a cross-sectional study carried out in 209 pregnant women from Eastern China, observed that an increase in selenium intake could reduce serum cadmium levels, improving foetal growth [25]. However, Horan et al., in a cohort analysis of 554 infants from the Randomised cOntrol trial of LOw glycaemic index diet to prevent macrosomia (ROLO) study (a randomized controlled trial conducted in Ireland of 800 secundigravid women with a previous macrosomic baby) showed that selenium was inversely related with the womb perimeter of newborns [26]. Also, in a Spanish study with a rather small sample size (*n* = 197 women), without data on SGA, no association was observed between selenium intake and newborn size [27].

Legumes, mainly lentils and chickpeas, have a high selenium content. In general, the content of selenium in most vegetables is low. The exception is garlic, which has 23 times the content of selenium of tomato or lettuce; also, green beans have almost three times the amount of selenium of other common vegetables [35, 36, 43]. In our results, both garlic and green beans reduced SGA risk. However, mushrooms, with a high selenium content, were unrelated to SGA risk in our population.

It would be very interesting to analyse whether diet can overcome the influence of obesity, malnutrition and other unfavourable conditions, such as low socioeconomic status and smoking. This can be addressed in future studies. Our sample had no statistical power to allow reliable analysis of these important variables; in our sample, obesity (BMI ≥ 30) was 8% (41 women in controls and 43 in cases), none of the patients were diagnosed with malnutrition and smoking women with a low socioeconomic status only comprised 10% of the population (*n* = 102). Regardless, the benefits of a healthy diet (with an adequate consumption of fruits, vegetables and legumes) should be widespread in the population.

There were no problems recruiting participants. The response rate was very high, so selection bias was not unlikely. The food frequency questionnaire (FFQ) had been validated and used previously with Spanish women [44, 45]. Mothers of normal newborns were density matched to cases to avoid the influence of season on the responses for certain types of foods. Also, women were matched by age to decrease generational effects on their food habits: younger women show a trend to have a more westernized diet than older ones, who adhere more to the Mediterranean diet [46].

In case-control studies certain problems of anamnestic bias cannot completely be ruled out, as women are aware of the newborn condition and this could influence their answers. During prenatal care no advice is given on taking specific foods, apart from avoiding raw meat and fish and reducing consumption of big fish (presumably with a higher content of mercury and other heavy metals). We asked women about any change in diet during pregnancy: 48.8% of cases reported increased vegetable intake vs. 46.7% of controls; the figures for fruit were 59.9% vs. 58.3 and 23.2% and 22.2% for legumes in cases and controls respectively. The percentages of change are roughly similar (although slightly higher in cases) between cases and controls and thus we believe that in the case of misclassification bias, it is most likely to be non-differential.

Confounding bias can also not be completely dismissed. Differences in weight gain, smoking and other variables were controlled for in multivariate analysis. This is a limitation inherent to most observational studies. Known risk factors for SGA help to explain only 30–40% of all cases, indicating that there is still much to be known about the epidemiology of this condition. We have also tried to control this bias by collecting data on the well-known risk factors for SGA and adjusting for them in the multivariable models.

Regarding specific legumes, a significant negative association was found with kidney beans; with other legumes, protective ORs were observed in the highest categories of intake, although none of them reached significance. When total legume intake was assessed, a significant negative trend was observed: the higher the intake, the lower the SGA risk. We have failed to identify any other report in which the intake of legumes is related to SGA or low birthweight and thus we cannot compare our results to other work.

## Conclusion

In conclusion, the intake of legumes and fruits seems to reduce the risk of SGA in Spanish women. Several vegetables containing selenium also reduced the incidence of SGA in our population. Nevertheless, the available information is scarce and more studies are needed on this subject.

## Additional file

Additional file 1: Questionnaire developed specifically for use in this study (DOC 721 kb)

## Abbreviations

AGA: Adequate for gestational age
aOR: Adjusted odds ratio
BMI: Body mass index
CIs: Confidence intervals
FFQ: Food frequency questionnaire
INMA: “INfancia and Medio Ambiente” (INMA, INfancy and Environment)
LGA: Large for gestational age
OR: Odds ratio
PH: Poniente Hospital
Q1: Quintile 1
Q2: Quintile 2
Q3: Quintile 3
Q4: Quintile 4
Q5: Quintile 5
ROLO: Randomised cOntrol trial of LOw glycaemic index diet to prevent macrosomia
SGA: small for gestational age
UGH: University of Granada Hospital
UH: Ubeda Hospital
UJH: University of Jaen Hospital
