# Supplementary material for: Maternal dietary consumption of legumes, vegetables and fruit during pregnancy, does it protect against small for gestational age?
Source: BMC Pregnancy Childbirth. 2018 Dec 11;18:486. doi: 10.1186/s12884-018-2123-4 (PMC6288906; doi:10.1186/s12884-018-2123-4)
Supplement: Supplementary file 1 — Questionnaire developed specifically for use in this study (DOC 721 kb) [file 12884_2018_2123_MOESM1_ESM.doc]

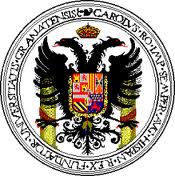


**Risk factors have a small newborn for gestational age (SGA)**

| **Initials of the interviewer (of the first name and surnames)** | |___|___|___| |
| --- | --- |
| **Place the Code that appears in the diet questionnaire (FFQ)** | |__|__|__|__|__| |
| **Hospital**  1-Clínico San Cecilio 3-Complejo H. de Jaén  2-Virgen de las Nieves 4-San Juan de la Cruz | |__| |
| **Initials of the mother (of the first name and surnames)** | |___|___|___| |
| **No. of clinical history** | |__|__|__|__|__|__|__|__|__| |
| **Type of mother: 0-Control: mother of AEG newborn**  **1-Case: mother of SGA newborn** | |__| |
| **No.of Case that is collected (correlative)** | |__|__| |
| **Case and control pair number** (The control mother is assigned the same number of the case with which it is matched) | |__|__| |
| **SOCIO-DEMOGRAPHIC DATA** |  |
| **Date of delivery** (day, month, year) | |__|__|-|__|__|-|__|__|__|__| |
| **Date of birth of the mother** (day, month, year) | |__|__|-|__|__|-|__|__|__|__| |
| **Marital status:** 1-Single 4-Divorced                                   2-Married 5-Separated                                   3-Couple 6-Widow | |__| |
| **Nationality:** 0-Spanish                                 1-Other; Specify:_________________ | |__| |
| **What level of education do you have?**        0-No studies 3-Secondary without final        1-Unfinished primary 4-Secondary completed        2-Elementary completed 5-University | |__| |
| **When does money come into the house monthly, approx.**        1- <1000 Euros        2- 1000-1999 Euros        3- 2000-2999 Euros  4- ≥3000 Euros | |__| |
| **Do you work outside the home? (**0. No 1. Yes)          If it says 'Yes', specify the task __________________ | |__| |
| **Race of women 0 -Caucasica**  **1-Other; specify ________________** | |__| |
| **Do you have any disease?**         0-No         1-Yes; Specify:______________________________ | |__| |
| **Did you usually take any medication before pregnancy?** 0-No       1-Yes; Specify:___________________ | |__| |
| **OBSTETRIC DATA** |  |
| **Number of pregnancies counting this one** | |__| |
| **Number of previous abortions** | |__| |
| **Number of children without counting this:** | |__|__| |
| **Has had a dead fetus (0-No 1-Yes)** | |__| |
| **Do you have any history of a child born preterm**? (0-No 1-Yes) | |__| |
| **Was one of your children SGA? (**0-No 1-Yes) |  |
| **Was one of your children born with low birth weight? (**0-No 1-Yes) | |__| |
| **Date of last birth** (day, month and year) | |__|__|-|__|__|-|__|__|__|__| |
| **Date of last menstruation** (day, month and year) | |__|__|-|__|__|-|__|__|__|__| |
| **Pregnancy sought (**0-No 1-Yes) | |__| |
| **Spontaneous pregnancy (without medical help): (**0-No 1-Yes) | |__| |
| **Have you done prenatal care?** (0-No 1-Yes) | |__| |
| **Number of visits for the control of this pregnancy:** | |__|__| |
| **Gestational week of the first visit:** | |__|__| |
| **Did you work (outside the housework) during pregnancy?** (0-No 1-Yes) | |__| |
| **How many weeks were you working during pregnancy?** | |__|__| |
| **How many hours a day have you stayed on average during pregnancy standing or walking?** | |__|__| |
| **Maternal weight before pregnancy (kg):** | |__|__|__|.|__| |
| **Maternal weight at the end of pregnancy (kg):** | |__|__|__|.|__| |
| **How much did you gain during pregnancy? (in kg)** | |__|__|.|__| |
| **Mother size (cm):** | |__|__|__| |
| **Have you taken this pregnancy?** |  |
| **Iron (**0-No 1-Yes) | |__| |
| *In what week of gestation he began to take* | |__|__| |
| *In what week of gestation he stopped taking* | |__|__| |
| *Remember the brand name of the drug?*  *Write it down ___________________________* |  |
| *Number of pills per day* | |__| |
| **Iodine (**0-No 1-Yes) | |__| |
| *In what week of gestation he began to take* | |__|__| |
| *In what week of gestation he stopped taking* | |__|__| |
| *Remember the brand name of the drug?*  *Write it down ___________________________* |  |
| *Number of pills per day* | |__| |
| **Folic acid** (0-No 1-Yes) | |__| |
| *In what week of gestation he began to take* | |__|__| |
| *In what week of gestation he stopped taking* | |__|__| |
| *Remember the brand name of the drug?*  *Write it down ___________________________* |  |
| *Number of pills per day* | |__| |
| **Polyvitamins** (0-No 1-Yes) | |__| |
| *In what week of gestation he began to take* | |__|__| |
| *In what week of gestation he stopped taking* | |__|__| |
| *Remember the brand name of the drug?*  *Write it down ___________________________* |  |
| *Number of pills per day* | |__| |
| ***Did you take any other medication as usual during pregnancy?*** *(0-No 1-Yes)*  *Specify ______________________________* | |__| |
| *In what week of gestation he began to take* | |__|__| |
| *In what week of gestation he stopped taking* | |__|__| |
| *Remember the brand name of the drug?*  *Write it down ___________________________* |  |
| *Number of pills per day* | |__| |
| **How many cigarettes / day did you smoke BEFORE pregnancy?** | |__|__| |
| **How many cigarettes / day did you smoke the first three months of pregnancy?** | |__|__| |
| **How many cigarettes / day did you smoke at the end of pregnancy?** | |__|__| |
| **On Saturdays and Sundays during pregnancy I used to take them at or before meals** |  |
| Any beer glass? (If it is "yes", inquire the number and write down) | |__| |
| Some wine glass? (If it is "yes", inquire the number and write down) | |__| |
| **On Saturdays and Sundays during pregnancy I used to take after meals ...** |  |
| some liquor glass? (If it is "yes", inquire the number and write down) | |__| |
| some combination (gin, rum, etc.)? (If it is "yes", inquire the nº) | |__| |
| **The working days during pregnancy used to take at meals or before them ...** |  |
| Any beer glass? (If it is "yes", inquire the number and write down) | |__| |
| Some wine glass? (If it is "yes", inquire the number and write down) | |__| |
| **The working days during pregnancy I used to take after meals ...** |  |
| some liquor glass? (If it is "yes", inquire the number and write down) | |__| |
| some combination (gin, rum, etc.)? (If it is "yes", inquire the nº) | |__| |
| **Have you used marijuana or cocaine during pregnancy?**  (0-No 1-Yes) Specify **_____________________** | |__| |
| **Have you had any health problems in pregnancy? (**0-No 1-Yes) | |__| |
| *Metrorrhagia greater than menstruation (0-No 1-Yes)* | |__| |
| *Metrorrhagia less than or equal to menstruation (0-No 1-Yes)* | |__| |
| *Hypertension (0-No 1-Yes)* | |__| |
| *Gestational diabetes (0-No 1-Yes)* | |__| |
| *Anemia (0-No 1-Yes)* | |__| |
| *Genitourinary infections 1st trimester (0-No 1-Yes)* | |__| |
| *Genitourinary infections 2nd trimester (0-No 1-Yes)* | |__| |
| *Genitourinary infections 3rd trimester (0-No 1-Yes)* | |__| |
| *Intrauterine infection (chorioamnionitis, etc.) (0-No 1-Yes)* | |__| |
| *Other (0-No 1-Yes)* | |__| |
| *Specify:_____________________* |  |
| **Were any of the following problems diagnosed during pregnancy?** (0-No 1-Yes) | |__| |
| *Placenta previa (0-No 1-Yes)* | |__| |
| *Low placenta (0-No 1-Yes)* | |__| |
| *Oligoamnios (0-No 1-Yes)* | |__| |
| *Myomas (0-No 1-Yes)* | |__| |
| *Was he tested for Pose or oxytocin test? (0-No 1-Yes)* | |__| |
| Was it positive? (0-No 1-Yes) | |__| |
| *Threat of abortion (0-No 1-Yes)* | |__| |
| *Threat of premature birth (0-No 1-Yes)* | |__| |
| *Cervical insufficiency (0-No 1-Yes)* | |__| |
| *Premature rupture of membranes (RPM) (0-No 1-Yes)* | |__| |
| *Week gestational. of the RPM in which it occurred:* | |__|__| |
| *Intrauterine growth restriction (0-No 1-Yes)* | |__| |
| *Another problem (0-No 1-Yes)* | |__| |
| *Specify ____________________________* |  |
| **DATA DELIVERY** |  |
| **The start of labor has been:**             1-Spontaneous 3-Induced             2-Stimulated 4- Cesarean section | |__| |
| **Type of delivery you have had**             1-Eutocic 2-Instrumental 3-Cesarean | |__| |
| **Gestational week in which the delivery took place:** | |__|__| |
| **Sex of the newborn (0-Woman 1-Man)** | |__| |
| **Weight of the newborn in grams** | |__|__|__|__| |
| **Cranial perimeter of the newborn (cm)** | |__|__| |
| **Length of the newborn (cm)** | |__|__| |
| **Score in the Apga test of the newborn to the minute of life:** | |__|__| |
| **Score in the Apga test of the newborn to the five minute of life:** | |__|__| |

**FFQ-Translation**

*Please, mark only one option for each food. For each food, mark the box that indicates the average consumption during the past year. We try take into account variation between summer/winter. For example, if you eat ice cream 4 times a week during the three months of summer, the average usage would be once a week per year.*

1. Whole milk (one cup, 200ml)
2. Half milk (one cup, 200ml)
3. Skim milk (one cup, 200ml)
4. Condensed milk (1 tablespoon)
5. Whipped cream or creamer (1/2 cup)
6. Milk shakes (one cup, 200ml)
7. Yogurt (1, 125g)
8. Low fat yogurt (1, 125g)
9. Petit suisse (1, 100g)
10. Curd or cottage cheese (half a cup)
11. Cream cheese or cheese slices (1 portion)
12. Other cheeses: cured or semi-cured (Manchego, Swiss cheese, etc) (50g)
13. White or fresh cheese (Burgos, goat cheese) (50g)
14. Custard, flan, pudding (1 cup, 200ml)
15. Ice cream (one)

Please, mark only one option for each food. One plate or portion of a 100-250g, except when otherwise indicated.

1. Chicken eggs (one)
2. Chicken or turkey WITH skin
3. SKINLESS chicken or turkey
4. Calf or cow meat
5. Pork meat
6. Lamb meat
7. Rabbit or hare
8. Liver
9. Other organs: brain, heart, etc.
10. Serrano ham
11. Baked ham (one slice)
12. Sausages (chorizo, salchichón, mortadela) (50g)
13. Sausages (50g)
14. Paté, foie-gras (25g)
15. Blood sausage (50g)
16. Hamburger (one)
17. Sobressada (50g), meatball (three)
18. Bacon, pancetta (50g)
19. White fish: whiting, hake, sea bream, grouper, flounder (1 plate, piece or portion)
20. Blue fish: sardines, tuna, mackerel, salmon (1 plate, piece or portion)
21. Cod
22. Salted or smoked fish: herring, salmon
23. Oysters, clams, mussels, etc (six)
24. Shrimp, lobster, crayfish
25. Octopus, calamari, squid, cuttlefish

One plate or portion of 250g, except when otherwise indicated.

1. Chard, spinach
2. Cauliflower, Brussel sprouts, broccoli
3. Lettuce, escarole, endive
4. Row tomato (one, 150g)
5. Carrots, pumpkin
6. Green beans
7. Eggplant, squash, cucumber
8. Peppers
9. Asparagus
10. Gazpacho
11. Other greens (borage, thistle)
12. French fries, home maid, bag (1 portion, 150g)
13. Baked or steamed potatoes (1 portion, 150g)

Please, mark only one option for each food. 1 plate or portion.

1. Orange, grapefruit (one), or mandarin (two)
2. Banana
3. Apple, pear
4. Strawberries (six, fruit salad)
5. Peach, apricot, nectarine
6. Cherries, plums (1 plate)
7. Figs
8. Watermelon (1 slice, 200-250g)
9. Melon (1 slice, 200-250g)
10. Canned fruits (2 cans)
11. Dates, dry figs, raisins (150g)
12. Almonds, peanuts, hazelnuts, walnuts (50g)
13. Olives (ten)
14. Avocado
15. Mangos, papaya
16. Kiwis

How many days a week you do it fruit as dessert?

Please, mark only one option for each food. 1 plate or portion of 60g dry weight.

1. Lentils
2. Chickpeas
3. Beans (pinto, white, black)
4. Peas
5. White bread (3 slices, 60g)
6. Wheat bread (3 slices, 60g)
7. Cereals (30g)
8. White rice (60g)
9. Pasta, noodles, macaroni, spaghetti (60g)
10. Pizza (1 slice, 200g)

Please, mark only one option for each food. 1 tablespoon or individual portion to deep, or dress salads, total:

1. Butter
2. Margarine
3. Olive oil
4. Sunflower oil
5. Corn oil
6. Lard
7. Other:

How often do you consume: fried foods at home, fried food at restaurants

At home, when frying, I use: olive oil, sunflower oil, corn oil, butter, margarine, others

Brand of olive oil that you usually use at home

Please, mark only one option for each food

1. Maria cookies (4-6 cookies, 50g)
2. Cookies with chocolate (4-6 cookies, 50g)
3. Packaged muffins (1-2 muffins)
4. Donuts (one)
5. Roll, croissant or other packaged pastries (one, 50g)
6. Home maid pastries or dessert
7. Cakes (one, 50g)
8. Churros (one portion, 100g)
9. Chocolates and truffles (30g)
10. Turron (1/8 bar)
11. Tea cookies, ice cream, mazapan (1 portion, 90g)

Please, mark only one option for each food

1. Glass of red wine
2. Glass of other type of wine
3. Glass of wine with meals
4. Beer (1 pitcher, 330ml)
5. Liquors: whisky, gin, cognac, licorice (1 glass, 50ml)
6. Carbonated beverages with sugar: coca cola, fanta (1 bottle, 200ml)
7. As above, but low calorie, diet beverages (1 bottle, 200ml)
8. Natural orange juice (1 glass, 200ml)
9. Other natural fruit juices (1 glass, 200 ml)
10. Bottled or canned fruit or vegetable juice (200ml)
11. Decaffeinated coffee (1 cup, 50ml)
12. Regular coffee (1 cup, 50ml)
13. Tap water (1glass, 200ml)
14. Bottled water (1 glass, 200ml)

List which brand of water bottle you usually drink.

Please, mark only one option for each food

1. Croquetas, bunuelas, empanadas
2. Soups and creams
3. Tomato sauce, ketchup (1 teaspoon)
4. Mayonnaise (1 teaspoon)
5. Spice: tabasco, red pepper
6. Salt (one pinch)
7. Sugar (one teaspoon)
8. Splenda
9. Jams (1 teaspoon)
10. Honey
11. Other foods frequently consumed

How frequently do you eat out?

Have you consumed vitamins and/or minerals (including calcium) regularly over the past year?

If yes, please list the brand

Normally, what do you do with the fat of cooked meat?

1. I eat it
2. I remove it

Do you try to consume much fiber?

Do you try to consume much fruit?

Do you try to consume much vegetables?

Do you try to consume much fish?

Do you usually eat snacks in between meals?

Do you follow a special diet? If yes, indicate the type of diet.

Do you avoid the use of butter?

Do you try to reduce your consumption of fat?

Do you try to reduce your consumption of meat?

Do you restrict salt in your diet?

Do you add sugar to some beverages?

Do you try to reduce consumption of sweets?
